# Role of circular RNAs in lung cancer

**DOI:** 10.3389/fgene.2024.1346119

**Published:** 2024-03-04

**Authors:** Maksat Babayev, Patricia Silveyra

**Affiliations:** Department of Environmental and Occupational Health, Indiana University School of Public Health Bloomington, Bloomington, IN, United States

**Keywords:** CircRNAs, circular RNA, lung cancer, lung adenocarcinoma, NSCLC, biomarkers

## Abstract

Lung cancer remains a global public health concern with significant research focus on developing better diagnosis/prognosis biomarkers and therapeutical targets. Circular RNAs (circRNAs) are a type of single-stranded RNA molecules that covalently closed and have ubiquitous expression. These molecules have been implicated in a variety of disease mechanisms, including lung cancer, as they exhibit oncogenic or tumor suppressor characteristics. Recent research has shown an important role that circRNAs play at different stages of lung cancer, particularly in lung adenocarcinoma. In this review, we summarize the latest research on circRNAs and their roles within lung cancer diagnosis, as well as on disease mechanisms. We also discuss the knowledge gaps on these topics and possible future research directions.

## 1 Introduction

Lung cancer is the leading cause of death among all cancers types, and remains among the top ten causes of death overall (Siegel et al., 2021). Lung cancer is classified into two main group types, non-small cell lung cancer (NSCLC) and small-cell lung cancer (SCLC), with NSCLC making up approximately 85% of all lung cancer cases (Siegel et al., 2021). The NSCLC group is further subdivided into three subtypes: lung adenocarcinoma (LUAD), squamous cell carcinoma (SCC), and large cell carcinoma (LCC), with LUAD making up most of the NSCLC cases (Siegel et al., 2022). While the latest developments in lung cancer diagnosis and treatment methods have improved the 5-year overall survival in lung cancer patients, there is still a need for identification of early diagnosis biomarkers, as well as more accurate prognostic biomarkers and more efficient therapeutic targets (Ettinger et al., 2021).

Circular RNAs (circRNAs) were discovered in the 1976 in the murine respirovirus (formerly known as Sendai virus) (Kolakofsky, 1976). The first discovery in humans, however, took place a decade later (Kos et al., 1986). Since their initial identification, numerous circRNAs have been detected in various organisms, including viruses, archaea, bacteria, and eukaryotic cells (Miao et al., 2021). Their presence has been linked to developmental phases, physiological states, and various diseases and conditions including cancer and cardiometabolic diseases (Giral et al., 2018; Chen et al., 2019; Di et al., 2019; Arthurs et al., 2022; Chen et al., 2022). This has paved the way for a new area of research focused on uncovering how circRNAs are formed, and their roles as fundamental components of gene expression processes. In the following sections, we summarize and discuss the roles that circRNAs have been shown to play in lung cancer initiation, progression, diagnosis, prognosis and response to therapeutics. We also provide a brief summary on the latest reported circRNAs and the potential roles that they can play in tackling lung cancer as oncogenes or tumor suppressors. Finally, we discuss future perspectives for lung cancer biomarker research including circRNAs.

## 2 CircRNA Biogenesis

**FIGURE 1:**
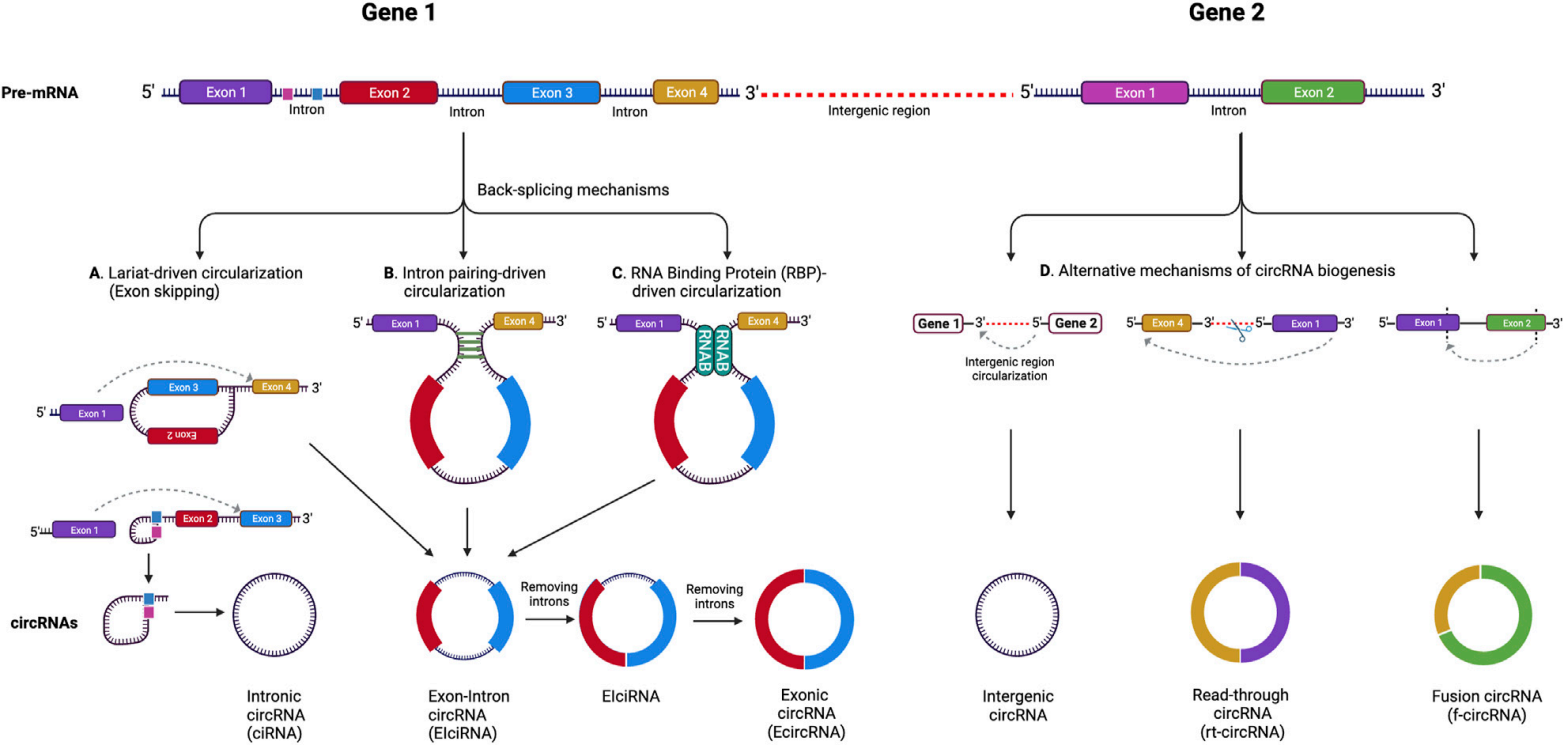
CircRNA biogenesis mechanisms. **(A)** Lariat-driven circularization (exon skipping), **(B)** intron pairing-driven circularization, **(C)** RNA binding protein (RBP)-driven circularization. Alternative circRNA biogenesis processes **(D)** leading to the formation of intergenic circRNA, rt-circRNA and f-circRNA. Adapted from (Pisignano et al., 2023), licensed CC-BY-4.0. Created with BioRender.com.

Circular RNAs are single-stranded, covalently closed RNAs that arise from protein-coding genes. During gene transcription, the RNA polymerase II transcribes the pre-mRNA, which then undergoes splicing at splicing sites known as spliceosomes. In this process, the circRNAs are generated when back-splicing takes place in parallel with canonical splicing, and the splice donor joins the splice acceptor to form a circular shaped RNA (Barrett et al., 2015). For a significant period of time, circRNAs were considered to be products of splicing errors (Nigro et al., 1991). The origin of the circRNAs was thought to be either from the lariat or from back-splicing (Zaphiropoulos, 1996; Jeck et al., 2013), however, a recent study proposed a unified model for circRNA biogenesis that includes intron and exon definition and back-splicing (Li L. et al., 2019) (Figure 1).

**FIGURE 2:**
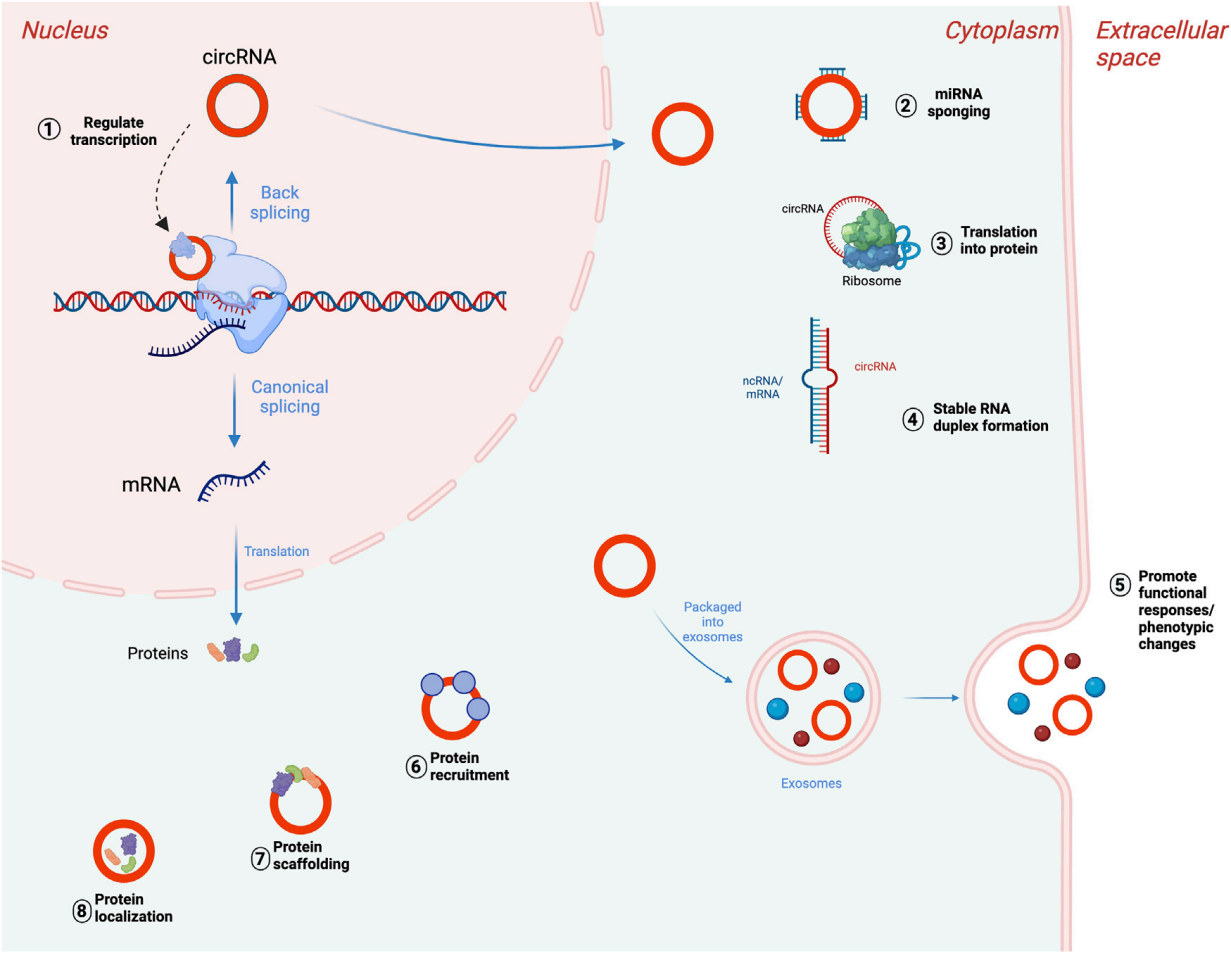
Biological functions of circRNAs. (1) Transcriptional regulation of parental genes, (2) posttranscriptional regulation through miRNA sponging, (3) translation of circRNAs into proteins or peptides. CircRNAs can also bind to mRNAs or lncRNAs and impact their stability (4), accumulate in exosomes to mediate cellular response (5), interact with RNA-binding protein (RBPs) (6), participate in protein scaffolding (7), and participating in protein localization pathways (8). Adapted from “DNA vs mRNA Transfection”, by BioRender.com (2022), https://app.biorender.com/biorender-templates, and (Pisignano et al., 2023), licensed CC-BY-4.0. Created with BioRender.com.

In addition, while the majority of circRNAs are made of exons from protein-encoding genes, they can also contain introns, intergenic regions, untranslated regions (UTRs), noncoding RNAs (ncRNAs) loci, and antisense locations of known transcripts (Caba et al., 2021) (Figure 1). CircRNA biogenesis can be modulated by RNA-binding proteins (RBPs) through RBP-driven circularization mechanism (Lyu and Huang, 2017; Zang et al., 2020). CircRNA generation can be facilitated via RBP binding to the introns close to the splicing sites (Conn et al., 2015). Some circRNAs can regulate their own expression post-transcriptionally by acting as a RBP sponge. For example, circMbl mediates its expression in a negative feedback loop manner by alternatively splicing its precursor RNA. (Begemann et al., 1997). RBPs are involved in circRNA splicing, folding, processing, localization, and stabilization through interaction with circRNA junctions (Janas et al., 2015). Figure 2 provides a summary of the known biological functions of circRNAs (Pisignano et al., 2023).

CircRNA molecules can be classified into several groups depending on their composition (Figure 1). These include: 1) exonic circRNAs (ecircRNAs), formed by exons exclusively and making up about 85% of all circRNAs, 2) circular intronic RNAs (ciRNAs), made of introns, and 3) exon-intron circRNAs (EIcircRNAs), formed by a combination of exons and introns (Zhou et al., 2018). There are also a few newly described circRNA categories including read-through circRNAs (rt-circRNAs), formed when an acceptor site at 5ʹ end of an exon binds to a donor site at a downstream 3ʹ end of an exon from the adjacent gene (read-through transcription), and fusion circRNAs (f-circRNAs), generated from chromosomal rearrangements such as translocations and deletions (Caba et al., 2021). These different types of circRNAs are found in various cellular compartments, with ciRNAs, EIcirRNAs, and f-circRNAs present in the nucleus, ecircRNAs found in cytoplasm and exosomes, and rt-circRNAs found in cytoplasm (Shang et al., 2019; He et al., 2021).

## 3 CircRNA functions

Even though circRNAs were previously considered non-coding RNAs (ncRNAs), there are several circRNAs that are translated and associated with cellular functions (Chen and Sarnow, 1995; Perriman and Ares, 1998; Pamudurti et al., 2017; Lei et al., 2020). The growing list of circRNA functions include transcriptional regulation (Conn et al., 2017), competing with endogenous RNAs by microRNA sponging (Hansen et al., 2013; Panda, 2018), modulating mRNA stability (Hansen et al., 2011), regulation of translation (Schuller and Green, 2018), translation into proteins (Zhou et al., 2020), and interaction with RNA binding proteins (Du et al., 2016).

### 3.1 Transcription regulation

A group of circRNAs containing introns are localized in nucleus and participate in transcriptional regulation of parental genes. EIciRNAs and ciRNAs play the cis-regulatory role by modulating parental genes. EIciRNAs perform the mentioned regulation via interaction with U1 small nuclear ribonucleoprotein (snRNP) forming the EIciRNAs-U1 snRNP complex. This complex further combines with polymerase II to modulate host gene transcription via its promoter region (Li et al., 2015; Wang et al., 2020). Another way circRNAs can generate *cis*-regulatory effects on parental genes is by accumulating at the transcriptional sites.

### 3.2 MiRNA sponging

MiRNAs are significant gene expression regulators at a post-transcriptional level that act by binding to sites within untranslated regions of messenger RNAs (Bartel, 2009). This underscores the importance of the most studied circRNA function, the miRNA sponging. The circRNA have several target sites to which miRNAs can bind, resulting in reduction of their regulatory function. It is also known that some circRNAs can act as a sponge for various miRNAs, acting as tumor suppressors or oncogenes (Chen et al., 2020). For example, the CDR1as, expressed in various human tissues, and lung carcinoma, has more than 60 binding sites and it acts as a competing endogenous RNA (ceRNA) for miR-7. As CDR1as is expressed, it leads to miR-7 activity inhibition with further increase in expression of miR-7 targets (Peng et al., 2015).

### 3.3 Protein interactions

Besides interacting with miRNAs, circRNAs have been reported to serve as a binding platform for argonaut proteins (Ago), proteins that are crucial in RNA silencing thorough mRNA cleavage or translation inhibition (Go and Mani, 2012). A study has demonstrated how RBPs are involved in miRNA recruitment and translation by circRNAs. The circBIRC6 was enriched in RBP Ago2 complex and regulated the pluripotency of human embryonic stem cells (hESCs) by combining with miR-34a and miR-145 (Yu et al., 2017). Involvement of Ago2 in the binding between circRNA-ZNF609 and miR-615-5p with further generation of similar effect on vascular endothelial dysfunction is another example of RBP participation in circRNA functional role (Liu et al., 2017). Additionally, with the help of RBPs, circRNAs can mediate mRNA translation or expression through methylation (Yang et al., 2017).

As circRNAs and linear RNAs come from common parental genes, the circRNAs with complete exons can be translatable, but in a cap-independent mechanism with the RBP assistance, different from conventional ribosome scanning mechanism (Du et al., 2017). CircRNAs have also demonstrated other interaction types with RBPs such as regulating RBPs by acting as sponges, competitively binding to RBPs, serving as super transporters, and platforms for RBP assembly (Hentze and Preiss, 2013; Du et al., 2017).

CircRNA can facilitate the interaction between two proteins by acting as a scaffold, but also can dissociate the interaction between proteins by binding to one. Additionally, circRNAs can block protein A from protein B by directly binding to only protein A. For example, CDR1as can block the tumor suppressor protein p53 from MDM2, thus preventing p53 ubiquitination and consequent DNA damage (Lou et al., 2020). MDM2 functions as an oncoprotein by hindering the transcriptional transactivation mediated by the p53 tumor suppressor. It not only guides p53 from the cell nucleus to the cytoplasm but also polyubiquitylates p53. The polyubiquitylated form of p53 undergoes swift degradation in the cytoplasm through the 26 S proteasome (Mendoza et al., 2014).

## 4 Role of circRNAs in Lung cancer

### 4.1 Role of circRNAs in lung tumorigenesis

Given the versatile roles the circRNAs can play within biological processes, recent studies have reported several mechanisms through which circRNAs are involved in lung tumorigenesis. These are summarized in Table 1. For example, a circRNAs named circHIPK3 is formed through circularization of the second exon of the homology domain-interacting protein kinase 3 (HIPK3) gene, and is highly expressed in cytoplasm of human lungs, brains, colon, stomach, heart and other organs (Zhou et al., 2021). This circRNA has been shown to activate the PI3K/AKT signaling pathway by sponging the miR-188-3p in lung cancer patients (Zhao et al., 2022).

**TABLE 1:** List of circRNAs as potential biomarkers of diagnosis, prognosis, and therapeutic targets for lung cancer.

CircRNA	Biomarker type	Cancer type	Expression	Role	Action mechanism	Ref.
circSATB2	Diagnostic	NSCLC	Up	Promoting cell proliferation, migration, and invasion of NSCLC cells	Promoting FSCN1 by sponging miR-326	Zhang et al. (2020)
circRNA-002178	Diagnostic	LUAD	Up	Promoting cancer cell immune evasion by promoting PD-L1 expression	Promoting PDL1/PD1 expression by sponging miR-34	Wang et al. (2020a)
circ_0047921	Diagnostic	NSCLC	Down	Promoting lung cancer progression	Binding to miRNA let-7g	Xian et al. (2020)
circFLNA	Diagnostic	NSCLC	Up	Promoting proliferation, migration, and invasion of cancer cells	Regulating XRCC1 and CYPA1 by sponging miR-486-3p	Pan et al. (2022)
hsa_circ_0023179	Diagnostic	NSCLC	Up	Associated with histological type, TNM stage, and metastasis		Zhang et al. (2023b)
circFOXK2	Diagnostic	NSCLC	Up	Promoting cell proliferation, migration, and invasion of NSCLC cells	Modulating IL-6 by sponging miR-149-3p	Xiang et al. (2023)
circUSP10	Diagnostic	NSCLC	Up	Associated with tumor size and TNM stage		Bai et al. (2023)
circFOXM1	Diagnostic/Prognostic	NSCLC	Up	Promoting cell proliferation and cell cycle progression	Suppressing of FAM83D by sponging miR-614	Yu et al. (2020)
circ_cMras	Diagnostic/Prognostic	LUAD	Down	Suppressing cell proliferation, migration, and invasion; promoting apoptosis	Acting through ABHD5/ATGL axis Using NF-κB signaling pathway	Zhou and Sun (2023)
has_circ_0000190 (C190)	Diagnostic/Therapeutic target	NSCLC	Up	Promoting cell proliferation and migration	Modulating EGFR-directed MAPK/ERK pathway by sponging miR-142	Ishola et al. (2022)
circCCDC134	Diagnostic/Therapeutic target	NSCLC	Up	Promoting cell growth, metastasis, and glycolysis	Regulating NFAT5 by sponging miR-625-5p	Tong et al. (2023)
circSCAP	Diagnostic/Therapeutic target	NSCLC	Up	Promoting cell proliferation, migration, and invasion	Upregulating SMAD by sponging miR-7	Zhang et al. (2023a)
hsa_circ_0088036	Prognostic	NSCLC	Up	Promoting cell proliferation, invasion, and migration	Activating TGFβ/Smad3/EMT signaling pathway via miR-1343-3p/Bcl-3 axis	Ge et al. (2023)
circHIPK3	Prognostic	LUAD	Down	Promoting cell proliferation, migration, and invasion; suppressing autophagy	Acting via miR-124-3p-STAT3-PRKAA/AMPKa axis	Chen et al. (2020)
circFGFR1	Prognostic	NSCLC	Up	Promoting anti-PD-1 resistance	Upregulating CXCR4 by sponging miR-381-3p	Zhang et al. (2019)
circFARSA	Prognostic	NSCLC	Up	Promoting cell proliferation, migration, and invasion of NSCLC cells	Upregulating B7-H3 by sponging miR-15a-5p	Nie et al. (2022)
circTUBGCP3	Prognostic	LUAD	Up	Promoting cell proliferation, colony formation	Suppressing cell proliferation and colony formation by sponging miR-885-3p	Yang et al. (2021)
circ-ANXA7	Prognostic	LUAD	Up	Promoting cell proliferation, migration, and invasion	Letting LAD1 promote proliferation and invasion through miR-331 sponging	Wang (2021)
circTP63	Prognostic/Therapeutic target	SCC	Up	Promoting cell proliferation	Upregulating FOXM1 by competitive binding to miR-873-3p	Cheng et al. (2019)
circXPO1	Prognostic/Therapeutic target	LUAD	Up	Promoting metastasis	Binding to IGFBP1 and stabilizing CTNNB1	Huang et al. (2020b)
circNDUFB2	Prognostic/Therapeutic target	NSCLC	Down	Suppressing tumor and favoring of antitumor immunity	Functioning as a scaffold forming complexes with TRIM25 and IGF2BPs for IGF2BPs ubiquitination	Li et al. (2021a)
circRNA_102231	Prognostic/Therapeutic target	NSCLC	Up	Promoting cell proliferation and invasion; associated with advanced TNM stage, lymph node metastasis, and poor survival	Promoting RBBP4 oncogene activity by sponging miR-145	Zong et al. (2018)
has_circ_0014130	Prognostic/Therapeutic target	NSCLC	Up	Promoting cell proliferation, invasion and inhibiting the apoptosis	Upregulating Bcl-2 by sponging miR-136–5p	Geng et al. (2020)
hsa_circ_0004689 (circSWT1)	Prognostic/Therapeutic target	NSCLC	Up	Promoting invasion, EMT, and migration; poor prognosis	Regulating SNAIL by sponging miR-370-3p	Long et al. (2023)
circCDR1	Prognostic/Therapy response	NSCLC	Up	Contributing to stemness and cis-platin chemoresistance	Targeting the miR-641/HOXA9 pathway by sponging miR-641	Zhao et al. (2020)
circ_10720	Prognostic/Therapy response	NSCLC	Up	Regulating EMT, promoting cell proliferation, migration, invasion, and inhibiting cell apoptosis		Martin et al. (2021)
CDR1-AS	Prognostic/Therapeutic target	LUAD	Up	Promoting chemoresistance to pemetrexed and cisplatin therapy	Promoting PTX and CDDP chemoresistance through EGFR/PI3K signaling pathway	Mao and Xu (2020)
FECR	Prognostic/Therapeutic target	SCLC	Up	Associated with lymph node metastasis, poor survival, response to chemotherapy	Inactivating tumor suppressor miR-584-3p leading to activation of ROCK1	Li et al. (2019a)
circ_0060967	Therapeutic target	NSCLC	Up	Promoting cell proliferation, migration, and invasion	Enhancing UBN2 expression by sponging miR-660-3p	Zhu et al. (2023a)
hsa_circ_0008305 (circPTK2)	Therapeutic target	NSCLC	Down	Suppressing EMT and cell invasion	Sponging of miR-429/miR-200b-3p that target TIFγ, and promoting EMT.	Wang et al. (2018)
hsa_circ_0007798 (circASK1)	Therapeutic target	LUAD	Down	Promoting chemosensitivity to gefitinib, suppressing gefitinib resistance	Activating ASK1/JNK/p38 pathway by encoding a protein ASK1-272a.a	Wang et al. (2021)
hsa_circ_0012673	Therapeutic target	NSCLC	Up	Promoting proliferation, motility, and EMT; suppressing apoptosis	Acting as ceRNA binding miR-320a and regulating LIMK1	Qin et al. (2020)
circ0000211	Therapeutic target	LUAD	Up	Promoting LUAD cell migration and invasion	Modulating HIF1-α expression by sponging hsa-miR-622	Feng et al. (2020)
circLIFR	Therapeutic target	NSCLC	Down	Suppressing cell proliferation, migration, and invasion	Inactivating PTEN/AKT pathway and regulating CELF2 by sponging miR-429	Wang et al. (2023b)
circRABL2B	Therapeutic target	NSCLC	Down	Suppressing cancer progression, cell stemness; promoting erlotinib sensitivity	Acting via MUC5AC/integrinβ4/pSrc/p53 axis	Lu et al. (2023)
circDLG1	Therapeutic target	NSCLC	Up	Promoting proliferation, migration, and invasion; suppressing apoptosis	Modulating miR-630/CENPF axis; regulating AKT/mTOR signaling and direct binding to miR-144	Chen and Xu (2023), Chen and Zhang (2023)
circ_0072088	Therapeutic target	NSCLC	Up	Promoting migration and invasion; suppressing apoptosis	Regulating the miR-1225-5p/WT1 axis	(Zhu et al., 2023a)
circ-ANXA7	Therapeutic target	NSCLC	Up	Promoting cell proliferation migration, invasion, and DDP resistance; suppressing apoptosis	Regulating CCND1 by sponging miR-545-3p	Yao et al. (2023)
circ-ZKSCAN1	Therapeutic target	LUAD	Up	Promoting DDP resistance, cell viability, migration, invasion, and glycolysis	Regulating TAGLN2 by sponging miR-185-5p	Yu et al. (2023)
circ_BLNK	Therapeutic target	NSCLC	Down	Suppressing DDP resistance, proliferation, migration, and invasion; promoting apoptosis	Regulating miR-25-3p/BARX2 axis	Liu et al. (2023)
circ-HSP90A	Therapeutic target	NSCLC	Up	Promoting cell proliferation migration, invasion, and immune evasion	Recruiting USP30 to stabilize HSP90A and stimulating STAT3 signaling, sponging miR-424-5p to PD-L1	Lei et al. (2023)
circ_0000376	Therapeutic target	NSCLC	Up	Promoting paclitaxel resistance and NSCLC tumorigenesis	Regulating KPNA4 by sponging miR-1298-5p	Hu et al. (2023)
circ-OXCT1	Therapeutic target	NSCLC	Up	Promoting cell proliferation migration, and invasion; suppressing apoptosis	Promoting SLC1A5 expression by binding to miR-516b-5p	Luo et al. (2023)
hsa_circ_0049657	Therapeutic target	NSCLC	Down	Promoting proliferation and migration; suppressing apoptosis		Ren et al. (2023b)
circFBXO7	Therapeutic target	NSCLC	Down	Acting as a tumor suppressor	Acting via circFBXO7/miR-296-3p/KLF15/CDKN1A axis	Wang et al. (2023c)
circ_0076305	Therapeutic target	NSCLC	Up	Regulating DDP resistance	Regulating ABCC1 expression by sponging miR-186-5p	Wang et al. (2023a)
circZCCHC6	Therapeutic target	NSCLC	Up	Promoting cell viability, cell cycle progression, migration, invasion, and EMT	Regulating LPCAT1 expression by sponging miR-433-3p	Guo et al. (2022)

Another circRNAs, circTP63, facilitates cell-cycle progression by acting as a competing endogenous RNA (ceRNA) and competitively binding to miR-873-3p (Cheng et al., 2019). Consequently, the binding prevents miR-873-3p from decreasing the levels of FOXM1 (forkhead box protein M1)*,* a proliferation-associated transcription factor, further upregulating CENPA and CENP genes, and thus promoting cell proliferation (Cheng et al., 2019). In a similar fashion, circFOXM acts as a ceRNA, suppressing the FAM83D (Family With Sequence Similarity 83 Member D) gene by sponging miR-614, and consequently promoting cell proliferation and cell cycle progression in NSCLC cells (Yu et al., 2020). In turn, the circFGFR1, which is significantly overexpressed in NSCLC tissues, acts as a tumor promoter by upregulating the C-X-C motif chemokine receptor 4 (CXCR4), a target gene for miR381-3p, and contributing to anti-PD-1 (Programmed cell Death Protein 1) resistance by sponging miR-381-3p (Zhang et al., 2019).

The B7-H3 (also known as CD276), member of the B7 immune checkpoint protein family, is overexpressed in cancer cells (Flem-Karlsen et al., 2018), thus promoting tumorigenesis. Contributing to this mechanism, circFARSA has been found overexpressed in cancer tissue and cell lines, leading to upregulation of B7-H3 via miR-15a-5p sponging (Nie et al., 2022). Similarly, circXPO1, a circRNA formed as a result of back-splicing of the well-established therapeutic agent XPO1 gene (Azizian and Li, 2020), is highly expressed in LUAD tissue. Its expression has been shown to correlate with worse survival in LUAD patients. The associated mechanism involves binding to IGF2BP1 (Insulin like Growth Factor 2 mRNA Binding Protein 1), stabilizing CTNNB1 (Catenin Beta 1), and consequently promoting LUAD progression (Huang et al., 2020).

Contrary to the previously mentioned circRNAs, elevated levels of circNDUFB2 inhibit tumor growth and metastasis in NSCLC cells by acting as a scaffold to enhance the interaction between the TRIM25 (Tripartite Motif Containing 25) and IGFBP2 (Insulin like Growth Factor Binding Protein 2) genes. Additionally, circNDUFB2 is recognized by RIG-I (Retinoic acid-Inducible Gene I) and leads to activation of the RIG-I-MAVS (Mitochondrial Antiviral Signaling protein) signaling cascade, with consequent immune cell recruitment into the tumor microenvironment (TME) (Li et al., 2021). Therefore, circRNAs can also have an inhibitory effect on lung tumorigenesis. In this regard, a circular RNA_ITCH (circ-ITCH) was reported to have significantly low expression in lung cancer tissue, and when expressed ectopically, it elevated levels of its parental cancer-suppressing gene, ITCH (Wan et al., 2016). Further analysis revealed that circ-ITCH enhanced ITCH expression by sponging miR-7 and miR-214, leading to suppression of the Wnt/*β*-catenin pro-oncogenic signaling pathway.

Another circRNA associated with LUAD is circTUBGCP3. Upregulation of this circRNA, or downregulation of miR-885-3p, was associated with pathological stage and poor survival in LUAD patients (Yang et al., 2021). Mechanistically, sponging of miR-885-3p by circTUBGCP3 prevents the miRNA from suppressing cell proliferation and attenuated the tumor-promoting effects of circTUBGGCP3. Circ-ANXA-7, which is highly expressed in LUAD tissue and cells, also promotes cell proliferation, migration, and invasion of LUAD cells, together with tumor growth (Wang, 2021). On the other hand, circ_0080608, which is under-expressed in tumor tissue, has been associated with suppressing lung cancer progression by regulating the miR-661/ADRA1A (Adrenergic Receptor Alpha 1A) pathway (Ren et al., 2023). Finally, circFOXK2 has been reported to modulate IL-6 via miR-149-3p sponging in NSCLC tissues and cells and thus promoting cell proliferation, migration, and invasion (Xiang et al., 2023).

Overexpression or activation of EGFR has been associated with poor prognosis in NSCLC (Kawai et al., 2005). A circRNA known as has_circ_0000190 (C190) has been found upregulated in both NSCLC clinical samples and cell lines, via a mechanism involving the EGFR pathway (Ishola et al., 2022). The C190 will be discussed more in the following sections having both prognostic biomarker and therapy target potentials.

### 4.2 CircRNAs and cancer stem cells

Stemness pertains to shared molecular mechanisms that enable stem cells to preserve their ability to produce more cells resembling themselves through self-renewal and to generate specialized offspring (Lagunas-Rangel, 2020). In this context, a small subset of cells emerging during cancer development, known as cancer stem cells (CSCs), exhibits both of these characteristics akin to normal stem cells (Yu et al., 2012). CSCs are considered the source of cancer cells, possessing the capacity to support tumor growth, re-establish a tumor upon transplantation into an immunodeficient animal host, instigate resistance to treatment, and contribute to metastatic dissemination (Peiris-Pages et al., 2016). Recent research indicates that the primary sources of these stem cells encompass (1) adult stem cells, (2) tumor cells, (3) differentiated cells, and (4) cell fusion (Feng et al., 2019).

Several investigations have suggested that the malfunction of various signaling pathways, including JAK/STAT, Hedgehog, WNT/β-catenin, Notch, PI3K/PTEN, and NF-κB, can facilitate the self-renewal and differentiation of cancer stem cells (CSCs) (Matsui, 2016). The pivotal aspect lies in the ultimate modification of gene expression patterns through pluripotent transcription factors like OCT4, NANOG, and SOX2, along with collaborating factors such as the KLF family, c-MYC, and Lin28 (Liu et al., 2013). Notably, an increasing body of evidence has showcased the participation of circular RNAs (circRNAs) in regulating signaling pathways associated with CSCs (Feng et al., 2019). These circRNAs function either as oncogenes or tumor suppressors, contingent upon the cell type, implying their potential as therapeutic targets and/or clinical biomarkers for the diagnosis, prognosis, or monitoring of the disease.

The ability of circRNAs to interact and sponge miRNAs and proteins leads to their involvement in CSCs role in cancer progression. For instance, a circ-CPA4 was reported to contribute to the regulation of stem growth, stemness, drug resistance and immune evasion in NSCLC. The regulation was demonstrated to take place via circ-CPA4/let-7 miRNA/PD-L1 axis (Hong et al., 2020). A recent study reported the role of circRNA hsa_circ_0003222, which accelerates stemness and progression of non-small cell lung cancer by sponging miR-527 (Li C. et al., 2021). Contribution to lung cancer stemness was reported for circ_POLA2 via miR-326/GNB1 axis, where the circ_POLA2 was highly expressed in lung cancer tissues and predicted a poor prognostic outcome (Fan et al., 2020).

The current body of evidence indicates that quiescent cancer stem cells (CSCs) play a role in cancer resistance to chemotherapy. Consequently, strategies solely focused on inhibiting CSC stemness may not be adequate to effectively counter post-chemotherapy recurrence. The tumor microenvironment (TME) is pivotal in regulating CSCs, maintaining their characteristics through various signals, and promoting the transition of non-stem cells to stem cell states (de Sousa e Melo et al., 2017). Targeting components of the TME appears to be a potentially more effective approach in overcoming treatment resistance compared to directly inhibiting CSC stemness. However, the complexity of immune cell heterogeneity across cell types poses challenges in precisely understanding CSC-immune cell interactions. Recently, the widespread use of single-cell RNA sequencing has enabled the identification of changing states in both CSCs and immune cells, shedding light on their interactions in different tumor contexts (Manni and Min, 2022). Further investigation is needed to explore the communication among these immune cells within the tumor microenvironment (TME), with the aim of advancing the development of immunotherapies that specifically target cancer stem cells (CSCs) more effectively.

### 4.3 Role of circRNAs in lung cancer diagnosis and prognosis

The circRNA circPVT1 is overexpressed in SCC tissue and serum from patients, and it promotes cell proliferation by acting as a ceRNA by sponging miR-30d and miR-30e, mitigating the suppressive effect of miRN-30d/e on cyclin F (CCNF) (Shi et al., 2021). A study that investigated role of circSATB2 in lung cancer reported its overexpression in NSCLC cells and tissue (Zhang et al., 2020). Furthermore, it was found that circSATB2 positively regulated fascin homolog 1 actin-bundling protein 1 (FSCN1) through miR-326 (Zhang et al., 2020). On the other hand, the circular RNA, hsa_circ_100395, was reported to act as a negative regulator of lung cancer development (Chen et al., 2018). Its decreased expression correlated with Tumor, Nodes, and Metastasis (TNM) stage and inversely correlated with poor prognosis. Another circRNA upregulated in LUAD and associated with the advanced TNM stage, lymph node metastasis and poor overall survival is circRNA_102231 (Zong et al., 2018).

One candidate for a prognostic biomarker role in lung cancer is has_circRNA_103809, which promotes cell proliferation *in vitro*, and its knockdown delays tumor growth *in vivo*. The mechanism proposed for this circRNA involves acting as a sponge of miR-4302, promoting ZNF121 expression, and consequently enhancing the proto-oncogene MYC protein levels in lung cancer cells (Liu et al., 2018). In NSCLC tissues, the significant upregulation of hsa_circ_0014130 is associated with proliferation and invasion. This circRNA also acts as a sponge of miR-136-5p leading to overexpression of its target gene Bcl-2 (Geng et al., 2020). More recently, the circRNA circFLNA has been found to promote lung cancer progression by acting as a sponge of miR-486-3p and thus regulating the XRCC1 (X-ray Repair Cross Complementing 1) and CYP1A1 (Cytochrome P450 Family 1 Subfamily A Member 1) genes (Pan et al., 2022).

A relationship between several circRNAs, including circ_10720 and epithelial-mesenchymal transition (EMT) process in NSCLC patients was reported in recent years (Martin et al., 2021). Furthermore, the circ_10720 was found to be overexpressed in some cell lines (HCC44, A549), and under-expressed in other (H23, H1299). Circ_10720 was overexpressed in NSCLC tissue, and its high levels were associated with shorter time to relapse (TTR).

Among the circRNAs that could have a prognostic biomarker potential for LUAD is circCRIM1 (hsa_circ_0002346). A study has concluded that this circRNA can act as a prognostic biomarker for LUAD survival as its downregulation in LUAD tissue has been significantly correlated with lymphatic metastasis and TNM stage (Wang et al., 2019). Indeed, circCRIM1 promotes the expression of leukemia inhibitory factor receptor (a tumor suppressor) by sponging miR-93 and miR-182 (Wang et al., 2019). Similarly, a circRNA that is upregulated in LUAD tissue and has prognostic potential is circ-ANXA7. Not only it promotes tumor progression as mentioned in the previous section, but also its high expression predicted poorer outcome for LUAD patients (Wang, 2021).

As liquid biopsies can serve as non-invasive biomarker sources, there is an interest in determining accurate circRNA biomarkers within them. Analysis of circRNA C190 in human blood showed association between high C190 levels with larger tumor size, worse histological type of adenocarcinoma, later cancer stage, more distant metastatic organs, extra-thoracic metastasis, poor survival, and prognosis (Luo et al., 2020). A study that investigated circRNA profile in Tumor-educated platelets (TEPs) has determined 411 circRNAs, out of 4,732 detected, to be significantly (*p*-value < 0.05) differently expressed in asymptomatic individuals compared to NSCLC patients. The nuclear receptor-interacting protein 1 (NRIP1) circRNA (circNRIP1) was selected as a potential biomarker shown to be significantly downregulated in platelets derived from NSCLC patient (D'Ambrosi et al., 2021). A programmed cell death ligand 1 (PDL1) and programmed death protein 1 (PD1) combine in a PD1/PDL1 pathway to control and maintain immune tolerance within the tumor microenvironment (TME) (Han et al., 2020).

A study focusing on circRNA role in LUAD tissue, found hsa_circRNA_002178 to be upregulated in cancer tissues, and that it enhanced PDL1 expression by sponging miR-34 to induce T-cell exhaustion. Furthermore, in plasma, circRNA-002178 was delivered into CD8^+^ cells by means of exosomes to promote PD1 expression (Wang J. et al., 2020). A study in Chinese population has shown that circ_0047921, circ_0056285, and circ_0007761 expression in serum exosomes could be used to distinguish early NSCLC cases from healthy controls (Xian et al., 2020). Furthermore, the circ_0047921 could distinguish early NSCLC cases from chronic obstructive pulmonary disease (COPD) controls, whereas combination of circ_0056285 and circ_0007761could distinguish between early NSCLC cases and tuberculosis controls.

A F-circEA, a circRNA generated by EML4-ALK fusion gene, is another potential liquid biopsy biomarker as it specifically exists in the plasma of EML4-ALK-positive NSCLC patients and contributes to tumor development by promoting cell migration and invasion (Tan et al., 2018). An investigation into NSCLC diagnostic potential of a circulating circular RNA hsa_circ_0023179 revealed that its higher expression was connected to histological type, TNM stage, lymph node metastasis, and distal metastasis in NSCLC tissue, serum and cells (Zhang et al., 2023). Moreover, it showed higher sensitivity and specificity when compared to traditional tumor markers.

While numerous circRNAs are introduced with theoretical potential of their diagnostic and prognostic value, an extensive amount of work lays ahead in investigating their reliability, precision and specificity. As of January 2024, the review of active clinical trials on circRNAs yields 12 registered clinical trials with 4 focusing on circRNA role in cancer (ClinicalTrials.gov). Common elements observed in the current clinical studies involve the research phase, specifically the discovery phase, and the necessity for subsequent validation in separate cohorts. Additionally, laboratory examinations are conducted to explore the molecular mechanisms underlying tumor-modifying behavior.

### 4.4 Role of circRNAs in lung cancer therapy

To date, a few circRNAs have been investigated for a potential role of a therapy target. For example, the C190 that could serve as a diagnostic biomarker, also could be considered as a therapy target.

More studies show the important role played by circRNAs in lung cancer therapy response. A study reported that the upregulation of CDR1-AS in LUAD tissue and cell lines was relevant to smoking history, T stage and neoadjuvant chemotherapy with pemetrexed (PTX) and cisplatin (CDDP) in LUAD patients (Mao and Xu, 2020). It was an independent prognostic biomarker therapy response, as it was highly expressed in LUAD tissues and cells (A549/CR) resistant to neoadjuvant chemotherapy with PTX and CDDP. Mechanistic investigation has revealed that CDR1-AS promoted PTX and CDDP chemoresistance through EGFR/PI3K signaling pathway. In another study, the circCDR1 and homeobox protein Hox-A9 (HOXA9) were overexpressed and miR-641 was low-expressed in cisplatin-resistant NSCLC cells (Zhao et al., 2020). The study showed that circCDR1 was regulating HOXA9 in NSCLC cells by acting as a miR-641 sponge, contributing to stemness and DDP chemoresistance. The circCRIM1 mentioned previously for its prognostic potential, was also shown to suppress the invasion and metastasis and in LUAD tissue, which makes it a potential therapeutic target (Wang et al., 2019). Treatment with growth factor receptor tyrosine kinase inhibitors is limited by the acquired resistance in the EGFR-mutant LUAD patients (Kobayashi et al., 2005). The circASK1 (hsa_circ_0007798) was shown to be significantly downregulated in gefitinib-resistant cells. It encodes for protein ASK1-272a.a, which is involved in ASK1/JNK/p38 signaling activation and mediates the chemosensitivity-inducing effect in of circASK1 in LUAD (Wang et al., 2021).

The hsa_circ_0008305 (circPTK2) and the transcriptional intermediary factor γ (TIFγ) have been found significantly downregulated in NSCLC cells undergoing the EMT induced by transforming growth factor β (TGF-β) (Wang et al., 2018). The TGF-β is highly expressed in NSCLCs and it promotes EMT and NSCLC cell invasion, whereas TIFγ acts as a tumor metastasis suppressor by regulating TGF-β/Smad signaling pathway (Dupont et al., 2005; Zhang et al., 2011; Xue et al., 2014). The circPTK2 overexpression was shown to suppress TGF-β-induced EMT as it sponges miR-429/miR-200b-3p, miRNAs that target TIFγ (Wang et al., 2018). These features and role make circPTK2 another potential therapeutical target candidate. On the other hand, circ_0060967, previously reported overexpressed in NSCLC tissue, was shown to promote cell viability, proliferation, migration, and invasion by sponging miR-660-3p and upregulating UBN2 (Zhu Z. et al., 2023). High expression of circ_0060967 implied poor prognosis and it could serve as a potential therapeutic target.

The overexpression of an otherwise under-expressed circLIFR in NSCLC tissue was found to suppress tumor progression and imped cell proliferation, migration, and invasion *in vivo* (Wang X. et al., 2023). A mechanistic investigation revealed circLIFR suppresses NSCLC progression by regulating CELF2 (CUGBP Elav-Like family Member 2) and inactivating PTEN (Phosphatase and Tensin homolog)/AKT (serine/threonine-protein kinase) signaling pathways by sponging miR-429. Low levels of circRABL2B in plasma exosomes could distinguish early stage lung-cancer patients, and it was revealed that circRABL2B counteracts lung cancer progression via MUC5AC (Mucin 5AC, Oligomeric Mucus/Gel-Forming)/integrin β4/pSrc/p53 axis, making the circRABL2B potential therapeutic target (Lu et al., 2023). An acceleration to cisplatin resistance in NSCLC patients by circ-ANXA7 was also recently reported (Yao et al., 2023). A mechanism behind this was concluded to be the regulation of CCND1 (Cyclin D1) by circ-ANXA7 via miR-545-3p sponging. A comparable finding on the role of circ-ZKSCAN1 on cisplatin resistance reported that circ-ZKSCAN1 promoted LUAD tumorigenesis and DDP resistance by regulating miR-185-5p/TAGLN2 (Transgelin 2) axis (Yu et al., 2023).

In SCLC chemoresistant cells, the circRNA cESRP1 (circular RNA epithelial splicing regulatory protein 1) was found significantly downregulated when compared to the parental chemosensitive cells (Huang et al., 2020). The same study identified cESRP1 as an important player in SCLC chemosensitivity by sponging miR-93-5p and inhibiting the TGF-β pathway (Huang et al., 2020). These findings suggest that cERP1 may serve as a prognostic and a potential therapeutic target in SCLC patients. Similarly, the abnormally upregulated Friend leukemia virus integration 1 (FLI1) is correlated with SCLC malignant phenotype (Li et al., 2017). A study that examined the role of FLI1 exonic circular RNAs (FECR) role as a new SCLC malignant driver, has revealed a positive association between the upregulated FECR1 and FECR2 and lymph node metastasis. It is also worth noting that serum exosomal FECR1 was associated with poor survival and chemotherapy clinical response (Li et al., 2019).

## 5 Discussion and future perspectives

CircRNAs have emerged as pivotal players in the intricate landscape of lung cancer. Our literature review confirmed that circRNA research has promising implications for enhanced diagnosis and novel therapies in the context of lung cancer, given the associations of circRNAs with various physiological processes and cell biology features of tumorigenesis, and their potential roles as biomarkers.

While significant strides have been made in unraveling the role of circRNAs in lung cancer, several knowledge gaps persist. First, the precise mechanisms by which circRNAs exert their regulatory functions in lung cancer remain incompletely understood. Elucidating the specific pathways and interactions involved could deepen our comprehension of their impact on disease progression. Additionally, the clinical utility of circRNAs as reliable biomarkers for lung cancer diagnosis and prognosis requires further validation across diverse patient populations. Standardized methodologies for circRNA detection and quantification are essential for ensuring reproducibility and comparability of results. Furthermore, the dynamic nature of circRNA expression patterns during different stages of lung cancer and their response to therapeutic interventions necessitate longitudinal studies to capture the temporal dynamics accurately. Lastly, translating our current knowledge of circRNAs into effective therapeutic strategies demands a more comprehensive understanding of their interactions with other cellular components and signaling networks.

While circRNAs frequently engage in multiple molecular processes across various tissues and diseases, it is important to note that targeting circRNAs for therapeutic purposes may result in unintended effects on non-cancerous cells and tissues, posing challenges for clinical application. Additionally, the identification of multiple microRNAs sponged by the same circRNAs in different cancer types implies that their impact on a particular cancer phenotype is likely influenced by the specific context. As the computing and artificial intelligence (AI) capabilities grow in hand with body of literature, AI algorithms can be used to analyze vast amounts of genomic and clinical data to identify novel circRNAs associated with lung cancer, aiding in the discovery of potential biomarkers for early diagnosis or prognosis. Machine learning models can also predict the functional roles of circRNAs by integrating diverse data sources, offering insights into their molecular mechanisms in lung cancer progression. Moreover, AI can facilitate the integration of multi-omics data, enabling a more comprehensive understanding of the complex interactions between circRNAs and other molecular components in lung cancer. This holistic approach may uncover previously unnoticed patterns and correlations, guiding researchers towards more targeted investigations. In drug discovery and therapeutic development, AI can expedite the identification of circRNAs that may serve as therapeutic targets. By predicting off-target effects and optimizing treatment strategies, AI can enhance the efficiency and safety of circRNA-targeted therapies.

The collaboration between the fields of data science, artificial intelligence (AI), and cancer research is crucial for unlocking the potential of AI in advancing cancer studies. The National Cancer Institute (NCI) can foster this collaboration by offering suitable funding opportunities and access to data sources. Additionally, facilitating connections between cancer researchers and AI experts, along with supporting the training and growth of a workforce skilled in AI, data science, and cancer, is essential. Drawing inspiration from the NCI–DOE collaboration, a series of workshops can form a community actively involved in pushing the boundaries of existing computational practices in cancer research and developing innovative computational technologies.

Bridging the mentioned gaps in mechanisms and technologies will undoubtedly enhance our ability to harness circRNAs for improved lung cancer diagnosis, prognosis, and treatment. Ongoing research may unveil the dynamic nature of circRNA expression during disease progression and treatment response. Longitudinal studies could provide insights into the temporal changes of circRNA profiles, offering valuable information for monitoring disease evolution and therapeutic efficacy. In essence, the future of circRNA research in lung cancer appears promising, with the potential for transformative contributions to early diagnosis, targeted therapies, and personalized treatment approaches.
